# β-Hemolysin, not *agrA* mutation, inhibits the hemolysis of α-hemolysin in *Staphylococcus aureus* laboratory and clinical strains

**DOI:** 10.1128/msphere.00673-23

**Published:** 2024-01-30

**Authors:** Lin Liu, Hemu Zhuang, Yanfei Wang, Yuexing Tu, Yunsong Yu, Yan Chen, Xueqing Wu

**Affiliations:** 1 Department of Infectious Disease, Sir Run Run Shaw Hospital, School of Medicine, Zhejiang University, Hangzhou, Zhejiang, China; 2 Laboratory Medicine Center, Department of Clinical Laboratory, Zhejiang Provincial People’s Hospital, Affiliated People’s Hospital, Hangzhou Medical College, Hangzhou, Zhejiang, China; 3 Key Laboratory of Microbial Technology and Bioinformatics of Zhejiang Province, Hangzhou, Zhejiang, China; 4 Regional Medical Center for National Institute of Respiratory Diseases, Sir Run Run Shaw Hospital, Zhejiang University School of Medicine, Hangzhou, Zhejiang, China; 5 Department of Respiratory and Critical Medicine, Sir Run Run Shaw Hospital, Zhejiang University School of Medicine, Hangzhou, Zhejiang, China; 6 Department of Critical Care Medicine, Tongde Hospital of Zhejiang Province, Hangzhou, Zhejiang, China; University of Nebraska Medical Center College of Medicine, Omaha, Nebraska, USA

**Keywords:** *Staphylococcus aureus*, *agrA*, β-hemolysin, α-hemolysin, δ-hemolysin

## Abstract

*Staphylococcus aureus* produces various hemolysins regulated by the Agr-QS system, except β-hemolysin encoded by the gene *hlb*. A classical laboratory *S. aureus* strain RN4220 displays only the β-hemolysin phenotype. It was suspected that the 8A mutation at the end of its *agrA* gene delayed the expressions of *hla* and *RNAIII*, then failed to express α- and δ-hemolysins. However, *hla* gene expression was detected at the later culture time without α-hemolysin phenotype, the reason for such a phenotype has not been clearly understood. We created *hlb* knockout and complementary mutants via homologous recombination in RN4220 and NRS049, two strains that normally produce β-hemolysin and carry *agrA* mutation. We found interestingly that the presence or absence of α-hemolysin phenotype in such strains depended on the expression of β-hemolysin instead of *agrA* mutations, which only inhibited δ-hemolysin expression. The hemolysis phenotype was verified by the Christie-Atkinson-Munch-Peterson (CAMP) test. Quantitative reverse transcription PCR was carried out to evaluate the relative gene expressions of *hlb*, *hla*, and *RNAIII*. The construction of mutants did not affect the *agrA* mutation status. We demonstrate that the absence of α-hemolysin in *S. aureus* RN4220 and NRS049 strains is attributed to their production of β-hemolysin instead of *agrA* mutation. Our findings broaden the understanding of the molecular mechanisms that control hemolysin expression in *S. aureus* that is crucial for the development of new therapeutic strategies to combat *S. aureus* infections.

**IMPORTANCE:**

α-Hemolysin is a critical virulence factor in *Staphylococcus aureus* and its expression is largely controlled by the Agr-QS system. Nonetheless, the hemolysis phenotype and the regulation of the Agr-QS system in *S. aureus* still hold many mysteries. Our study finds that it is the expression of β- hemolysin rather than the *agrA* mutation that inhibits the function of the α-hemolysin in an important *S. aureus* strain RN4220 and a clinical strain presents a similar phenotype, which clarifies the misunderstood hemolytic phenotype and mechanism of *S. aureus*. Our findings highlight the interactions among different toxins and their biological roles, combined with QS system regulation, which is ultimately the true underlying cause of its virulence. This emphasizes the importance of considering the collaborative action of various factors in the infection process caused by this significant human pathogen.

## OBSERVATION

*Staphylococcus aureus* is an opportunistic pathogen that can cause a range of infections in humans, from mild skin infections to severe systematic infections (1, 2). The pathogenicity of *S. aureus* is largely attributed to the presence of virulence factors. For example, *S. aureus* would lysis erythrocytes by producing different hemolysins, such as α-, β-, and δ-hemolysins (3). The expressions of α- and δ-hemolysins are regulated by the staphylococcal Agr-QS system (4, 5). The effector of this system is the *RNAIII* (6), which stimulates transcription of the downstream *hla* gene, encoding α-hemolysin, while the *hld* gene encoding δ-hemolysin is located within the *RNAIII* locus (7). The *agr* locus consists of two transcriptional units driven by P2 and P3, which are activated by the regulator AgrA (8). Differently, the expression of β-hemolysin in some *S. aureus* strains is dependent on the presence or absence of prophage insertion in its encoding gene *hlb* (9) and its regulation awaits further study (10). β-Hemolysin, also known as hot-cold hemolysin, has enhanced hemolytic activity below 10°C and appears to function as a sphingomyelinase (SMase) that binds to the sphingomyelin of endothelial cells and erythrocytes (11–13). We know relatively little about β-hemolysin compared to other hemolysins, which has promoted us to study the biological function of this staphylococcal toxin.

**Fig 1:**
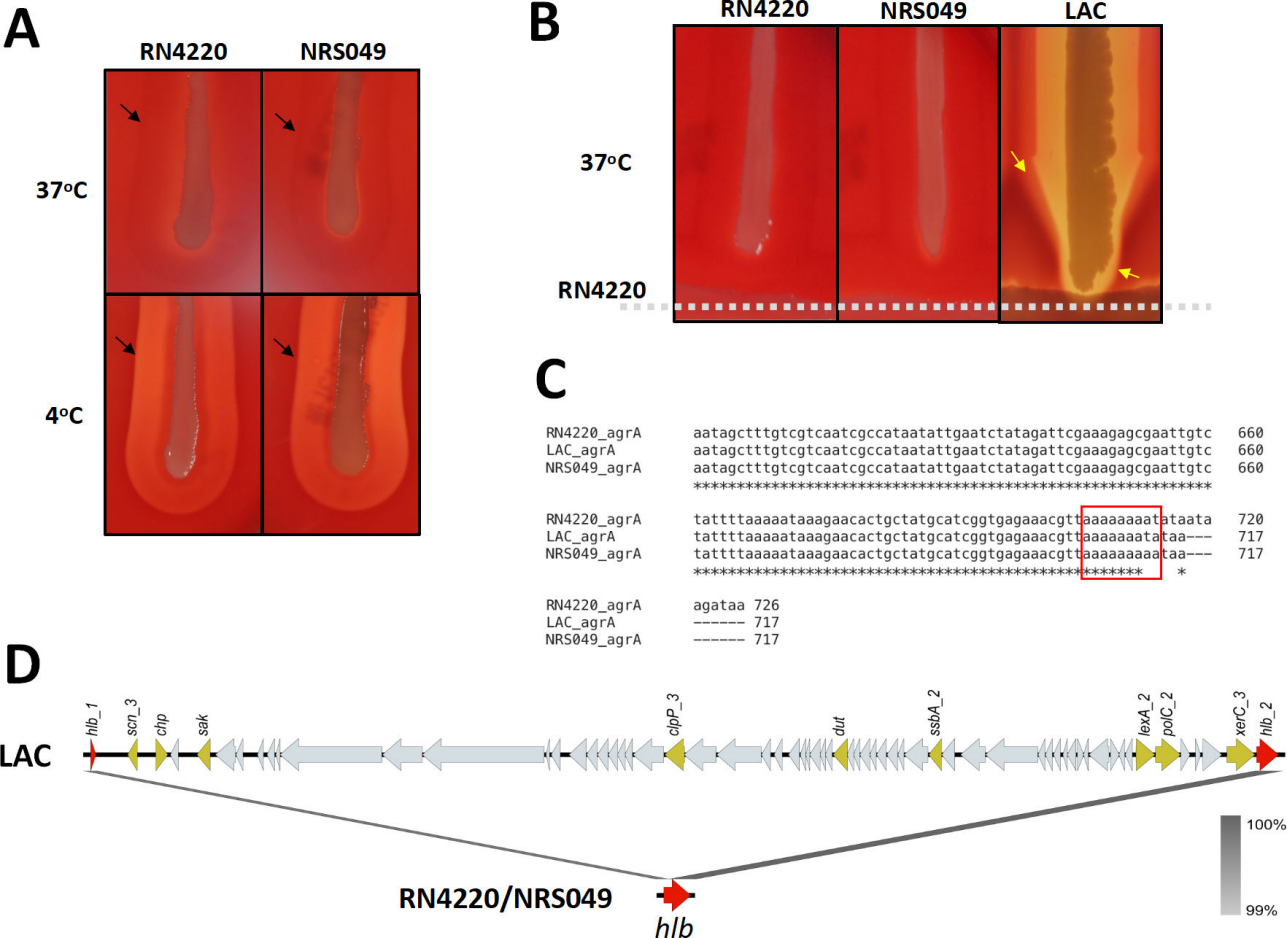
Hemolysis phenotype of *S. aureus* strains. (**A**) Hemolysis phenotype of tested laboratory strain RN4220 and clinical strain NRS049 on sheep blood agar plate under 37°C and 4°C. (**B**) The CAMP tests for RN4220, NRS049, and LAC strain. Strains were tested against RN4220 to evaluate their hemolytic activities: β-hemolysin phenotype occurred in RN4220 and NRS049, α-hemolysin (yellow arrows marked decreased hemolysis when crossing the β-hemolysin from underlined RN4220) and δ-hemolysin (yellow arrows marked enlarged hemolysis when crossing the β-hemolysin) phenotypes occurred in LAC strain. (**C**) The sequences of gene *agrA* in LAC, RN4220, and NRS049 were compared via https://www.ebi.ac.uk/Tools/msa/clustalo/. Different from the normal *agrA* gene in LAC, an 8A and a 9A mutation at the site of 713 in RN4220 and NRS049 was observed, respectively. (**D**) β-Hemolysin encoding gene *hlb* was compared between LAC and RN4220/RNS049 using Easyfig 2.2.5 (https://mjsull.github.io/Easyfig/) and depicted a prophage insertion in the *hlb* gene of LAC that causes its failure to produce *β*-hemolysin.

We choose two *S*. *aureus* strains, RN4220 (a classic laboratory strain) and NRS049 (a clinical strain), both of which only produce β-hemolysin, and a α- and δ-hemolysin producer, the USA300 strain LAC (Fig. 1A and B). We analyzed the major hemolysin regulator encoding gene *agrA* in these strains and found an 8A mutation in RN4220 and a 9A mutation in NRS049 at the site of 713 (Fig. 1C). Previous reports have shown that mutations in *agrA* result in a late transcription of *RNAIII* and the absence of α- and δ-hemolysins in *S. aureus* (14, 15). However, it is not clear why the α-hemolysin phenotype was not observed even though the *hla* message was always detected about 1 h after the expression of *RNAIII*. The β-hemolysin phenotype was not seen in strain LAC due to a prophage insertion in its *hlb* gene that was visualized via Easyfig 2.2.5 (16) (Fig. 1D).

**Fig 2:**
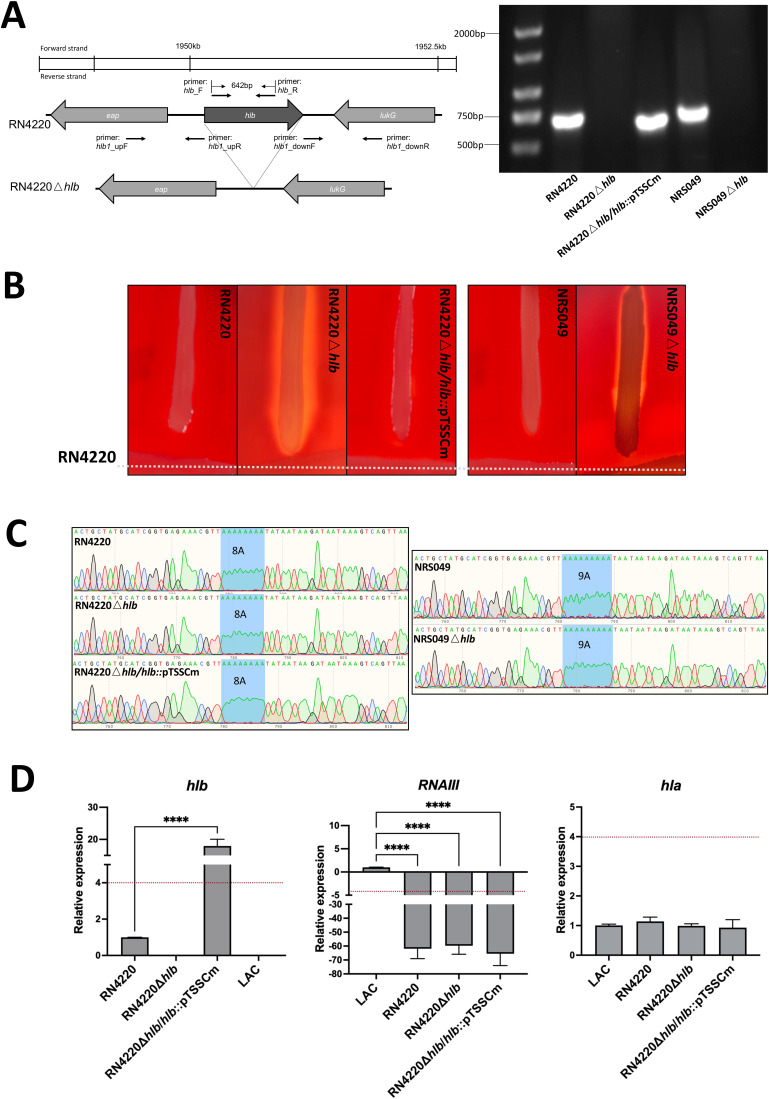
β-Hemolysin inhibits α-hemolysin phenotype, rather than *agrA* 8A/9A mutations, in *S. aureus* that express both. (**A**) Schematic representation of the *hlb* gene deletion and gel electrophoresis confirmation of *hlb* knockout and complementary construction. (**B**) The CAMP test results presentation. Strains were tested against RN4220 to evaluate their hemolytic activities: α-hemolysin phenotype occurred in RN4220Δ*hlb* and NRS049Δ*hlb* and disappeared in RN4220Δ*hlb/hlb::*pTSSCm. (**C**) Sanger sequencing results of the *agrA* gene, where the 8A/9A mutation was confirmed to continue to exist in all mutants. (**D**) The relative expression levels of *hlb*, *RNAIII,* and *hla* in LAC and RN4220 derivatives. The red dashed line indicates the cutoff of a fourfold change in each panel, which was considered a significant increase or decrease. “****” represents *P* < 0.0001 for one-way analysis of variance (ANOVA) tests. The comparison was conducted against the gene expression level in RN4220 for *hlb* and in LAC for *RNAIII* and *hla*.

When we knocked out the *hlb* gene in strains RN4220 and NRS049, we expected no hemolysis on the sheep blood agar plate (Fig. 2A). Surprisingly, as shown in Fig. 2B of the CAMP (Christie-Atkinson-Munch-Peterson) test (17), a strong α-hemolytic halo was observed around all *hlb* knockout strains, including RN4220Δ*hlb* and NRS049Δ*hlb*. Furthermore, the *α*-hemolytic halo disappeared in the *hlb* complementary strain RN4220Δ*hlb/hlb*::pTSSCm. As previously reported, the 8A mutation at the end of the *agrA* gene can cause a delay in *RNAIII* expression and thus may ultimately result in a failure of *α*- and δ-hemolysin translations (14). To ascertain if the *agrA* gene was affected during our *hlb* knockout process, we amplified the *agrA* gene of all wild type, *hlb* mutants, and *hlb* complementary strains by PCR and verified it by Sanger sequencing. The results showed that 8A and 9A mutations of *agrA* continue to exist in all tested strains (Fig. 2C). We found that the presence of α-hemolytic halo depended on the lack of expression of β-hemolysin instead of the mutations that occurred at the end of *agrA* gene. To further confirm our findings, we also conducted quantitative reverse transcription PCR for *hlb*, *RNAIII,* and *hla* genes in all RN4220 derivatives and LAC at 2 h of incubation. According to the *hlb* expression pattern in RN4220 wild type, Fig. 2D demonstrates that RN4220Δ*hlb* and LAC did not exhibit any detectable *hlb* activity, whereas LAC and RN4220Δ*hlb/hlb*::pTSSCm displayed high levels of *hlb* expression. As expected, the expression level of *RNAIII* was found to be significantly reduced in strain RN4220, regardless of the presence of the *hlb* gene, while the expression of *hla* remained unaffected. This was observed by comparing the expression levels in strain RN4220 to those in LAC. Demonstrating the mutations at the end of gene *agrA* resulted in a loss of δ-hemolysin gene expression, while α-hemolysin expression remained unaffected in all tested strains.

In summary, *S. aureus* strains with the 8A or 9A mutation at the end of the *agrA* gene fail to express the δ-hemolysin (*RNAIII*) but not α-hemolysin. The inhibition of α-hemolysin phenotype in such strains is due to the expression of β-hemolysin, which is an Mg^2+^-dependent neutral sphingomyelinase that hydrolyzes sphingolipids into phosphatidylcholine and ceramide (12). Since *S. aureus* α-hemolysin is a cell membrane pore-forming toxin, causing an outflow of intracellular material and leads to cell death (18), the binding of α-hemolysin to the human cell membrane is the first step of this process, which significantly relies on the presence of sphingolipids (19). Removal of sphingomyelin from the human airway epithelial cells plasma membrane will completely abrogate the damaging effect of α-hemolysin. Therefore, when β-hemolysin is present and consumes sphingolipids, α-hemolysin lacks the binding targets and hence fails to form pores on the erythrocyte membrane. This explains that the presence of α-hemolytic halo is rarely observed in strains that produce β-hemolysin, regardless of their *agrA* mutation status, unless the expression of α-hemolysin is sufficiently high (20).

The mystery of why some β-hemolysin-producing *S. aureus* strains (RN4220) do not show α-hemolysin phenotype despite having a functional *hla* gene has puzzled researchers for some time. This study has solved this mystery by demonstrating that the presence of β-hemolysin inhibits α-hemolysin phenotype activity regardless of the strains’ *agrA* mutation status and results in a phenotype with only β-hemolysin phenotype when the expression of δ-hemolysin is inhibited in *S. aureus*. Our findings broaden the understanding of the molecular mechanisms that control hemolysin expression in *S. aureus*, which is crucial for the development of new therapeutic strategies to combat *S. aureus* infections.

### Mutant construction

Mutants were constructed as described previously with slight modification (21). Briefly, allelic substitutions were made by amplifying approximately 1 kb regions upstream and downstream of the target gene *hlb* and cloning them into plasmid pKOR1. The plasmid was first transformed into *S. aureus* RN4220 and then into NRS049. To integrate the plasmids, RN4220 (pKOR1-*hlb*) and NRS049 (pKOR1-*hlb*) cultures were grown in TSB containing 10 µg/mL chloramphenicol (TSB_Cm10_) at 30°C and were transferred to fresh TSB_Cm10_ at 43°C overnight. The deletion mutants were verified using PCR and Sanger sequencing. For the complementary strain construction, the wild-type and mutant derivatives of *hlb* were cloned into pTSSCm and transferred into strain RN4220Δ*hlb*. Briefly, the *hlb* gene was amplified using PCR, cloned into the *SpeI*- and *NdeI*-digested plasmid pTSSCm, and transformed into *S. aureus* RN4220, which was then transformed into strain RN4220Δ*hlb* by electroporation. These plasmids conferred tetracycline resistance, and tetracycline was added to growth cultures at a concentration of 12.5 µg/mL. The parental *Escherichia coli* DH5α and RN4220Δ*hlb* strains carrying the empty plasmid pTSSCm were used as controls. The *hlb* complementary strain was only constructed for strain RN4220, due to NRS049 being a multi-drug resistant strain that lacks selection marker for the *hlb* complementary screening. The strains and plasmids used in this study are listed in Table 1 and the primers used are listed in Table S1.

**TABLE 1:** Strains and plasmids used in this study

Strain or plasmid	Description	Reference
Strains		
RN4220	*agrA* 8A mutation; β-hemolysin	(22)
NRS049	*agrA* 9A mutation; β-hemolysin	(23)
LAC	No mutations in the *agrA*; δ- and α-hemolysins	(24)
RN4220Δ*hlb*	RN4220 with a deletion of the *hlb* gene; α-hemolysin	This study
RN4220Δ*hlb/hlb*::pTSSCm	RN4220Δ*hlb* strain complementing pTSSCm; β-hemolysin	This study
NRS049Δ*hlb*	NRS049 with a deletion of the *hlb* gene; α-hemolysin	This study
*E. coli* DH5a	Host strain for construction of recombination plasmids	
Plasmids		
pKOR1	*E. coli/S. aureus* shuttle vector plasmid	(21)
pTSSCm	*E. coli/S. aureus* shuttle vector plasmid	(25)

### CAMP test

According to the interaction between the hemolysins of *S. aureus* that β-hemolysin inhibits the lysis of α-hemolysin but enhances the lysis of δ-hemolysin (8, 26), we performed a CAMP test (17) to confirm the hemolysin phenotype of each *S. aureus* strain. For instance, we determined all strain hemolysin types by cross-hatching them vertically on sheep blood agar plates with RN4220 (14). The degree of hemolysis at the cross indicates phenotypes of different hemolysins: the same as for RN4220 is determined as β-hemolysin, the attenuated hemolysis is determined as α-hemolysin, and the enhanced hemolysis is determined as δ-hemolysin.

### RT-qPCR

Cultures of *S. aureus* strains LAC, RN4220, RN4220Δ*hlb,* and RN4220Δ*hlb/hlb::*pTSSCm were grown to a density of 50 Klett units (1.5 × 10^8^ cells/mL), diluted 10-fold times, and grown again to 50 Klett units. After 2 h of culture, samples were harvested for total RNA extraction using the E.Z.N.A. Total RNA Kit (OMEGA, Switzerland) according to the manufacturer’s protocol. The quality of extracted RNA was assessed using a NanoDrop spectrophotometer (Thermo Fisher Scientific, USA). Thereafter, the total RNA was reverse-transcribed to cDNA using the PrimeScript RT reagent Kit (TaKaRa, Japan), and RT-qPCR was then carried out using the TB Green Fast qPCR Mix (TaKaRa, Japan) via Roche LightCycler 480 Real-Time PCR System. Primers used for RT-qPCR assays are listed in Table S1. The relative quantitation of mRNA expression was normalized to the constitutive expression of the housekeeping *gyrB* and *rho* genes and calculated by the comparative CT (2^−ΔΔCT^) method. A fold change over 4 was considered a significant increase or decrease.

### Statistical analysis

For the RT-qPCR test results, the relative expression of each tested gene that was decreased or increased over fourfold would be considered as biologically significant down- and upregulation. Meanwhile, we also conducted one-way ANOVA tests to compare the differences between groups for each gene expression experiment, where *P* < 0.0001 was considered statistically significant. All analyses were performed using GraphPad Prism V9.5.0. All experiments were repeated at least three times.

## SUPPLEMENTAL MATERIAL

Table S1 msphere.00673-23-s0001.docx Primers used in the study.

ASM does not own the copyrights to Supplemental Material that may be linked to, or accessed through, an article. The authors have granted ASM a non-exclusive, world-wide license to publish the Supplemental Material files. Please contact the corresponding author directly for reuse.
