# Supplementary material for: β-Hemolysin, not agrA mutation, inhibits the hemolysis of α-hemolysin in Staphylococcus aureus laboratory and clinical strains
Source: mSphere. 2024 Jan 30;9(2):e00673-23. doi: 10.1128/msphere.00673-23 (PMC10900901; doi:10.1128/msphere.00673-23)
Supplement: Table S1 — Primers used in the study. [file msphere.00673-23-s0001.docx]

**Supplementary Table 1.** Primers used in the study

| **Primer name** | **Sequence (5’-3’)** | **Reference** |
| --- | --- | --- |
| *hlb1*_upF | TGACGTTGAGCCTCGGAACCGGTACCCGGTAATACCTCTATTTGATTC | This study |
| *hlb1*_upR | AGTTATTAGTTAGTTGAGCACATTATCACTCCTTTTATATAGCTTAC |  |
| *hlb1*_downF | TATAAAAGGAGTGATAATGATGCTCAACTAACTAATAACTTGC | This study |
| *hlb1*_downR | CGACGGCCAGTCTTAAGCTCGGGCCCGCAAAAGGTACAATTGGTAGTG |  |
| *hlb2*_upF | TGACGTTGAGCCTCGGAACCGGTACCAATACCTCTATTTGATTC | This study |
| *hlb2*_upR | GTTAGTTGAGCACATTATCACTCCTTTTATATAGCTTAC |  |
| *hlb2*_downF | GGAGTGATAATGTGCTCAACTAACTAATAACTCGC | This study |
| *hlb2*_downR | CGACGGCCAGTCTTAAGCTCGGGCCCAGCAAAAGGTACAATTGGTAGTG |  |
| *agrA*_F | GCTGATAATGCAGACAATG | This study |
| *agrA*_R | GCTATACAGTGCATTTGCT |  |
| RNAIII_RT_F | CCTAGATCACAGAGATGTGATGG | (1) |
| RNAIII_RT_R | AATACATAGCACTGAGTCCAAGG |  |
| *hla*_RT_F | GTCATTTCTTCTTTTTCCCAATCG | (2) |
| *hla*_RT_R | CACGTATAGTCAGCTCAGTAACA |  |
| *hlb*_F | TAGTTGGTGCACTTACTGAC | This study |
| *hlb*_R | ACTCTGGAGTGCCTTTATTG |  |
| *gyrB*_F | GTCGAAGGGGACTCTG | (3) |
| *gyrB*_R | GCTCCATCCACATCGG |  |
| *rho*_F | GAAGCTGCTGAAGTCG | (3) |
| *rho*_R | CGTCCATACGTGAACCC |  |
| *hlb_*RT_F | GCCAAAGCCGAATCTAAG | (4) |
| *hlb*_RT_R | CGAGTACAGGTGTTTGGT |  |

**Supplemental References**

1. Yan J, Liu Y, Gao Y, Dong J, Mu C, Li D, Yang G. 2015. RNAIII suppresses the expression of LtaS via acting as an antisense RNA in *Staphylococcus aureus*: RNAIII suppresses the expression of LtaS. J Basic Microbiol 55:255–261.

2. Liu L, Wang B, Yu J, Guo Y, Yu F. 2022. NWMN2330 May Be Associated with the Virulence of Staphylococcus aureus by Increasing the Expression of hla and saeRS. IDR Volume 15:2853–2864.

3. Sihto H-M, Tasara T, Stephan R, Johler S. 2014. Validation of reference genes for normalization of qPCR mRNA expression levels in Staphylococcus aureus exposed to osmotic and lactic acid stress conditions encountered during food production and preservation. FEMS Microbiol Lett 356:134–140.

4. Zhang H, Zheng Y, Gao H, Xu P, Wang M, Li A, Miao M, Xie X, Deng Y, Zhou H, Du H. 2016. Identification and Characterization of Staphylococcus aureus Strains with an Incomplete Hemolytic Phenotype. Frontiers in Cellular and Infection Microbiology 6.
